# The Development, Applications, and Future Directions of Nutritional Literacy Scales: A Scoping Review

**DOI:** 10.3390/nu18101616

**Published:** 2026-05-20

**Authors:** Hanqian Shao, Zeying Huang

**Affiliations:** Institute of Food and Nutrition Development, Ministry of Agriculture and Rural Affairs, Beijing 100081, China; 821012450760@caas.cn

**Keywords:** nutritional literacy, scale development, measurement tools, application scenarios, health promotion, scoping review

## Abstract

Background: Nutritional literacy is a core competency for promoting healthy dietary behaviors and preventing nutrition-related chronic diseases. Standardized scales are essential for rigorous measurement and evaluation, yet the field exhibits substantial heterogeneity in concepts and measurement approaches. Methods: We systematically searched five major databases, namely Web of Science, PubMed, Scopus, Embase, and CINAHL, from their inception to October 2025. Evidence was compiled on the conceptual evolution, domain structure, scoring logic, population-specific applicability, and application scenarios of nutritional literacy scales. Results: A total of 14 nutritional literacy scales developed between 2005 and 2025 were included in the review. The structure and measurement content of these scales have progressively expanded, evolving from an early focus on basic reading and numeracy skills to become multidimensional assessment tools encompassing knowledge, skills, and behavioral practices. The target population has broadened from the general adult population to include multiple special groups, while application regions have extended from high-income Western countries to developing regions, including China and Turkey, and assessment methodologies have progressively shifted from single tests to blended objective–subjective approaches, with most scales demonstrating sound reliability and validity. These instruments are now employed for screening, intervention evaluation, dietary behavior mechanism research, and analysis of chronic disease risk. The reviewed studies indicate that nutritional literacy is generally positively correlated with healthy dietary behaviors, nutrition labeling utilization, and related health outcomes. Conclusions: Although nutritional literacy scale research has advanced with regard to conceptualization, measurement design, and applications, major gaps remain, including fragmented dimensional structures, insufficient standardization, inadequate cultural adaptation, and limited longitudinal evidence. Future work should prioritize a unified assessment framework, stronger tools for special and vulnerable populations, digital innovations for scalable measurement, and interdisciplinary and cross-national collaboration to enhance quality, practicality, and comparability and to support global nutrition promotion and public health policy.

## 1. Introduction

As one of the primary behavioral risk factors for preventable diseases and premature mortality worldwide [1], unhealthy diets and malnutrition contribute to nearly one-third of the global burden of disease [2]. Achieving global targets for healthy diets depends not only on food system capacity and policy interventions but also, more critically, on individuals’ ability to understand, evaluate, and act on nutrition information. Nutritional literacy is therefore recognized as a key factor driving changes in dietary behavior [3]. It helps individuals identify nutrition-related risks, optimize dietary patterns, and adopt healthy behaviors, thereby playing an important role in preventing chronic diseases such as overweight and obesity, cardiovascular disease, and diabetes [4]. Therefore, research on the measurement, assessment, and intervention of nutritional literacy is not only important for advancing global health strategies and achieving population-wide health but also provides a solid theoretical foundation and practical basis for disease prevention and health promotion. Although the relevant body of research continues to increase, nutrition literacy measurement tools still differ in terms of theoretical foundations, dimensional structures, target populations, scoring methods, and reliability and validity testing, which limits the comparability and integration of findings across studies. In addition, although the target populations and application scenarios of nutrition literacy research continue to expand, systematic summaries of the development, measurement characteristics, and current applications of nutrition literacy scales remain limited. Therefore, conducting a scoping review is necessary to comprehensively map the evolution, core characteristics, and application scenarios of these scales, thereby providing a reference for tool selection, cross-cultural adaptation, and intervention studies for nutrition literacy.

## 2. Materials and Methods

This study employed a scoping review methodology, with literature retrieval, screening, and reporting conducted according to the official PRISMA-ScR guidelines (PROSPERO registration No. CRD420251266854) [5]. Given the considerable differences among existing studies in terms of scale types, study populations, cultural backgrounds, and application purposes, a scoping review is more suitable for summarizing the distribution of research in this field and identifying gaps. This study aims to review the development process, measurement characteristics, applicable populations, and application scenarios of nutrition literacy scales, rather than evaluate the effects of specific interventions or conduct a simple synthesis. Therefore, a scoping review design was chosen. Inclusion criteria were established based on the PCC framework. The population comprised individuals of any age, gender, geographical location, and health status. The concept focused on nutrient literacy measurement tools, encompassing their development, translation, adaptation, psychometric evaluation, and application in studies examining dietary behavior and health-related outcomes. No restrictions were imposed on the context, covering healthcare, community, school, hospital, and other relevant research or practice settings. Literature screening and data extraction were conducted independently by two researchers. Prior to formal screening, the research team prescreened selected studies to standardize the inclusion and exclusion criteria. Reasons for exclusion during full-text screening were recorded by one researcher and reviewed by another. Following data extraction, both researchers cross-checked the results; discrepancies were resolved through discussion and re-examination of the original texts.

### 2.1. Information Sources and Retrieval Strategy

**Figure 1:**
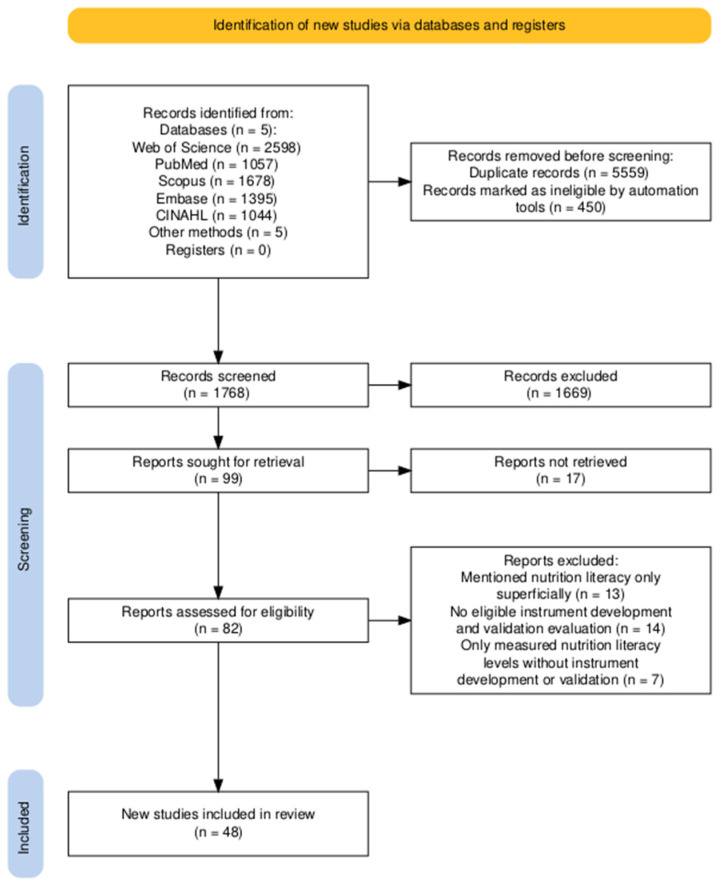
Flowchart for the selection of sources of evidence.

The search databases included Web of Science, PubMed, Scopus, Embase, and CINAHL, with search fields limited to titles, abstracts, and keywords. The search covered studies published from 1963 to December 2025, and no explicit restrictions were imposed on languages during the retrieval process. The retrieval strategy leveraged two complementary search formulas, both centered on core concepts related to nutrition literacy and combined with nutrition and diet-related terminology. The first formula identifies research on nutrition literacy measurement tools by layering psychometric and scale-related terms onto core concepts to retrieve studies on scale development, translation, cultural adaptation, reliability, and validity testing. The second set identifies empirical research linking nutritional literacy to health behaviors and health outcomes. It builds upon the same core concepts by incorporating health behavior and outcome-related terms to retrieve applied studies where nutritional literacy serves as a key or explanatory variable. Each database query was adjusted for equivalence according to platform syntax. In addition to database searches, citation tracking was conducted by screening the reference lists of relevant articles. Through citation tracking, we identified five further publications, all of which were theses. Full texts were obtained via Google Scholar, with detailed records provided as “Other methods” in Figure 1.

### 2.2. Inclusion and Exclusion Criteria

Included studies encompass original research directly related to measurement tools for nutritional literacy and related concepts, covering scale development, translation or cultural adaptation, and assessments of validity, reliability, and other psychometric evidence, as well as applied research explicitly using named scales in observational or interventional studies. Excluded studies included research measuring only nutritional knowledge rather than the concept of nutritional literacy; studies failing to explicitly name scales or lacking key measurement information; Chinese literature and conference abstracts, news reports, newspapers, errata, and records without full text; and literature unrelated to the research topic. Reasons for exclusion at the full-text stage are documented and presented in Figure 1.

### 2.3. Literature Screening and Data Extraction

The records retrieved from the databases were exported to Excel, and information related to titles, abstracts, authors, publication year, journal name, and DOI was extracted. Duplicates were identified primarily by title and manually verified using the authors, year, journal name, and DOI; 5559 duplicate records were removed. Before screening, 450 records that were missing titles, abstracts, or other key information were removed, leaving 1768 records for title and abstract screening. At this stage, 1669 records were excluded, including irrelevant studies, reviews, conference abstracts, and conference papers. A total of 99 reports, including five theses identified through citation tracking, were used in full-text retrieval. Seventeen reports were unavailable, leaving 82 for eligibility assessment. After full-text review, 34 reports were excluded because they only briefly mentioned nutrition literacy, lacked eligible tool development or validation, or only measured general nutrition literacy without examining tool development, validation, or associations with health behaviors or outcomes. Finally, 48 studies were included. The screening process is shown in Figure 1.

### 2.4. Data Tabulation Process

After obtaining full-text studies for inclusion, the research team created data tables in Excel, organizing findings into three categories: the evolution of conceptual definitions of nutrition literacy (Table 1), the development and psychometric characteristics of nutrition literacy measurement tools (Table 2) and evidence on the application of named scales in empirical studies and their associations with health behaviors and health outcomes (Table 3). Extracted content included basic bibliographic information (authors, year, and publication source), study population and setting, scale name and version, scale structure and scoring method, and psychometric evidence (reliability, validity). For scale application studies, additional details were recorded: research domain, target population, statistical methods, key findings, and annotations on how the scale was used in the study and its corresponding behavioral or health outcome variables. Data extraction and synthesis were grounded in full-text information, with reference to original methodology and results sections when necessary to verify scale structure, scoring, and psychometric evidence. Findings are summarized in Table 2 and Table 3.

## 3. Results

### 3.1. The Evolution of the Concept of Nutritional Literacy

The conceptual definition of nutritional literacy has transformed alongside the evolution of health communication paradigms. Around 2006, influenced by literacy research within public health, the definition of nutritional literacy was initially centered on the use of basic literacy and numeracy skills to decode food labeling information [6]. With the rise in global digital nutrition assessment tools (such as MyPyramid) in 2007, scholars such as Neuhauser (2007) incorporated quantitative dietary assessment into the definition of nutrition, emphasizing information-processing capabilities within digital environments [7]. After 2008, international nutrition research shifted towards dietary decision-making. Silk (2008) positioned nutritional literacy as a key factor in sound nutritional decision-making [8], while Zoellner (2009) and Watson (2013) further established the behavioral logic chain of acquisition, comprehension, and application through empirical studies on chronic disease management [9,10]. In 2014, inspired by the global dissemination of Nutbeam’s three-tiered health literacy model, Guttersrud (2014) proposed a framework for nutritional literacy with three tiers: functional, interactive, and critical [11,12]. Consequently, the concept evolved from a purely cognitive dimension to one encompassing critical thinking and the capacity for sound decision-making (Table 1).

**Table 1:** Summary of the concept of nutritional literacy.

Definition of Nutritional Literacy	Article
Nutritional literacy, as a specific form of health literacy, requires both general literacy and computational skill.	(Blitstein&Evans, 2006 [6])
Adequate health literacy and nutrition literacy require an individual not only to read well but also to understand health and nutrition concepts and to have basic quantitative skills.	(Neuhauser et al., 2007 [7])
Nutrition literacy can be defined similarly to health literacy as the degree to which individuals can obtain, process, and understand the basic health (nutrition) information and services they need to make appropriate health (nutrition) decisions.	(Silk et al., 2007 [8])
Nutrition literacy may be defined as the degree to which people have the capacity to obtain, process, and understand basic nutrition information.	(Zoellner et al., 2009 [10])
‘Nutrition literacy’ can mean the extent to which people access, understand and use nutrition information.	(Watson et al., 2013) [9]
Nutrition literacy can be defined as ‘the capacity to obtain, process and understand nutrition information and the materials needed to make appropriate decisions regarding one’s health. There are three cumulative levels of nutrition literacy: ‘functional’, ‘interactive’ and ‘critical’. A ‘critical dimension’ of nutrition literacy is ‘the ability to critically assess nutritional information and dietary advice’.	(Guttersrud et al., 2014 [11])

Sources of information compiled by the authors based on relevant literature.

### 3.2. Advances in Research on Nutritional Literacy Scales

This study reviewed 14 nutritional literacy scales developed between 2005 and 2025, sourced from multiple countries and regions, including the United States, Norway, Japan, Uganda, Turkey, Australia, and China. Regarding dimensional structure, the number of dimensions in these scales gradually expanded from one or two in earlier versions to a maximum of six. The measurement content evolved from reading comprehension and numerical calculations to comprehensive assessments encompassing knowledge, skills, and behavioral practices. In terms of target populations, the scales’ coverage progressively broadened from the general adult population to include individuals with chronic diseases, older adults, children, adolescents, pregnant women, and lactating women. Regarding measurement methods, early instruments primarily employed contextualized test items and knowledge assessment questions. Later tools predominantly adopted a hybrid model combining knowledge questions with Likert scales, with some scales further incorporating dietary frequency questionnaires. In terms of reliability and validity, Cronbach’s α coefficients for the scales ranged from 0.59 to 0.96, and construct validity was supported through exploratory or confirmatory factor analysis. Notably, there are seven localized versions of the NLAI (Nutrition Literacy Assessment Instrument), making it the most extensively cross-culturally adapted tool series among the included scales (as indicated in Table 2).

### 3.3. Progress in Research on the Application of Nutritional Literacy Scales

Research on the application of nutrition literacy scales encompasses seven major thematic domains: dietary behavior, comprehension and utilization of nutrition labeling, nutritional and health status, nutrition information acquisition, sleep patterns, dietary patterns among chronic disease populations, and quality of life. The geographical distribution of studies is extensive, encompassing countries and regions including China, Turkey, Palestine, Hong Kong, Iran, Chile, Azerbaijan, the United States, and Canada. Target populations span adults, adolescents, preschool children, and individuals with chronic conditions. Regarding tool utilization, the NLS (Nutritional Literacy Scale), CNL (Critical Nutrition Literacy Scale), and NLit (Nutrition Literacy Assessment Instrument) demonstrate the broadest cross-domain application, while the NVS (Newest Vital Sign), EINLA (Evaluation Instrument of Nutrition Literacy on Adults), and NLit-S (Nutrition Literacy Assessment Instrument in Spanish) are selectively employed within specific research domains. Statistical methods primarily comprised Pearson correlation analysis, chi-square tests, regression analysis, and analysis of variance, with some studies incorporating factor analysis and mediation effect testing. Key findings are highly consistent across research domains, with nutrient literacy levels showing significant positive correlations with dietary behaviors, nutrition labeling comprehension, nutritional health status, and quality of life. Furthermore, parenting styles mediate the conversion of nutrient literacy into dietary behaviors among preschool children, while nutrient literacy interventions exhibit spillover effects on health behaviors such as sleep quality (as indicated in Table 3).

**Table 2:** Summary of existing studies on nutritional literacy measurement instruments.

Scale Name	National Source	Propose Time	Dimension	Applicable Population	Measurement Question Type	Reliability and Validity Test	Internal Consistency	Scale Expansion
NVS (Weiss et al., 2005) [13]	United States	2005	Reading Comprehension; Numerical Calculation	Chronic disease patients	Scenario-based test questions	The Pearson correlation coefficients: TOFHLA = 0.59; REALM = 0.41; S-TOFHLA = 0.60	Cronbach’s α = 0.76 (English version); Cronbach’s α = 0.69 (Spanish version)	-
NLS (Diamond et al., 2007) [14]	United States	2007	Reading and writing skills	Adult	Knowledge measurement questions;	The Pearson correlation coefficients S-TOFHLA = 0.61	Cronbach’s α = 0.84	Spanish-NLS (Coffman et al., 2012) [15]; NLS-Gr (Michou et al., 2019) [16]
NLQ (Kjøllesdal, 2009) [17]	Norway	2009	Functional nutrition literacy; Interactive nutrition literacy; Critical nutrition literacy	Adult	Knowledge measurement questions; Likert scale	EFA: KMO = 0.77; Bartlett’s: *p* < 0.001; R^2^ = 0.52	Cronbach’s α = 0.86	-
NLQ-JP (Aihara&Minai, 2011) [18]	Japan	2011	Eating habits	Elderly	Knowledge measurement questions; Task-based comprehension questions; Likert scale	The Pearson correlation coefficients: S-TOFHLA = 0.65; NVS = 0.16	Cronbach’s α = 0.86	-
CNL (Blegen, 2011) [19]	Norway	2011	Critical nutrition literacy	Adolescents	Likert scale	EFA: KMO = 0.77, Bartlett’s *p* < 0.001, R^2^ = 0.45	Cronbach’s α = 0.90	-
NLAI (Gibbs, 2012) [20]	United States	2012	Nutrition & Health; Energy Sources in Food; Food Label & Numeracy; Household Food Measurement; Food Groups; Consumer Skills	Chronic disease patients	Knowledge measurement questions; Knowledge comprehension questions; Image True or False Questions; Label Calculation problem; Food Classification Questions	CFA: CFI > 0.90: RMSEA < 0.05	Cronbach’s α = 0.96	NLit-Bca (Gibbs&Ellerbeck, et al., 2016) [21]; NLit-P (Gibbs&Kennett, et al., 2016) [22]; NLit(Gibbs et al., 2017) [23]; NLit-S (Gibbs, et al., 2018) [24]; NLit-IT (Vettori et al., 2021) [25]; NLit-Br (Sarkis et al., 2022) [26]; CHI-Nlit (Zhang et al., 2025 [27])
ANLS (Bari, 2012) [28]	Uganda	2012	Functional nutrition literacy; Interactive nutrition literacy; Critical nutrition literacy	Adolescents	Likert scale	-	Cronbach’s α = 0.59	-
EINLA (Cesur, 2014) [29]	Türkiye	2014	Reading comprehension; food groups, portion sizes; reading food labels; simple numeracy	Adult	Knowledge measurement questions, knowledge comprehension questions; Food Classification Questions; Image True or False Questions; Label Interpretation Questions	EFA: KMO = 0.76; Bartlett *p* < 0.05; R^2^ = 0.36	Cronbach’s α = 0.75	-
e-NutLiT (Ringland et al., 2016) [30]	Australia	2016	Locating information; Calculations; Comparing nutrition information panels; Impact of endorsement logos	Adult	Food Calculation Problems; Knowledge measurement questions; Label Interpretation Questions	The Pearson correlation coefficients: NVS = 0.73;	-	-
CNL-E (Naigaga et al., 2018) [31]	Norway	2018	Functional nutrition literacy; Interactive nutrition literacy; Critical nutrition literacy	adolescents	Likert scale	CFA: CFI = 1.00; NNFI = 0.999, RMSEA = 0.054; SRMR = 0.009	Cronbach’s α = 0.90; Person Separation Index= 0.88	-
FNLQ-SC (Liu et al., 2021) [32]	China	2021	Food and nutrition knowledge; Access to and planning for food; Selecting food; Preparing food; Eating	School-age children	Knowledge measurement questions; Likert scale	EFA: RMSEA = 0.070; GFI = 0.838; AGFI = 0.813	Cronbach’s α = 0.698	-
NLQ-E (Aihemaitijiang et al., 2022) [33]	China	2022	Knowledge and understanding; lifestyle and dietary behavior; skills	Elderly	Likert scale; Food Consumption Frequency Question; Knowledge measurement questions	CFA: RMSEA = 0.045; PCFI = 0.776; PNFI = 0.759	Cronbach’s α = 0.678	-
NLAI-P (Zhou et al., 2022) [34]	China	2022	Basic knowledge and ideas; Lifestyle and dietary behaviors; Basic skills	Pregnant women	Knowledge measurement questions; Likert scale	EFA: KMO ≥ 0.74; χ^2^/df = 1.82; RMSEA = 0.046	Cronbach’s α = 0.82	-
FNLQ (Zhang et al., 2022) [35]	China	2022	Food and nutrition Knowledge; Access to and planning and selecting for food; Preparing and marking food; Eating	Adult	Knowledge measurement questions; Likert scale	EFA: KMO = 0.923; RMSEA = 0.048; AGFI = 0.876	Cronbach’s α = 0.893	-
Nutrition Literacy Measurement Scale for Chinese Adults (Zhang et al., 2022) [36]	China	2022	Knowledge; Understanding; Obtaining; Applying; Interactive; Critical	Adult	Likert scale	CVI = 1.0; Kendall’s W > 0.83; authority coefficient = 0.90 ± 0.06	-	-
NLAI-L (Li et al., 2023) [37]	China	2023	Knowledge; Behavior; Skill	Lactating women	Knowledge measurement questions; Likert scale	CVI = 0.98; CVR = 0.96; χ^2^/df = 2.28; RMSEA = 0.057	Cronbach’s α = 0.84	-
NLQ-PSC (Wen et al., 2025) [38]	China	2025	Knowing about food; Knowing about the characteristics of food; Selecting food; Eating behaviors; Eating safely; Physical activities	preschool children	Knowledge measurement questions; Likert scale	Children’s Questionnaire: χ^2^/df = 1.378; RMSEA = 0.043; GFI = 0.901; AGFI = 0.875, PNFI = 0.546; Parent Questionnaire: χ^2^/df = 1.318; RMSEA = 0.048; GFI = 0.914; AGFI = 0.873	Children’s Questionnaire: Cronbach’s α = 0.660 Parent Questionnaire: Cronbach’s α = 0.628	-

Note: Compiled by the authors based on relevant literature. Abbreviations: NVS, The Newest Vital Sign; NLS, The Nutritional Literacy Scale; Spanish-NLS, The Spanish Nutrition Literacy Scale; NLQ, The Nutrition Literacy Questionnaire; NLQ-JP, The Nutrition Literacy Questionnaire for Japanese; CNL, The Critical Nutrition Literacy Scale; NLAI, The Nutrition Literacy Assessment Instrument; NLit-BCa, The Nutrition Literacy Assessment Instrument for Breast Cancer; NLit-P, The Nutrition Literacy Assessment Instrument for Parents; NLit, The Nutrition Literacy Assessment Instrument; NLit-S, The Nutrition Literacy Assessment Instrument in Spanish; NLit-IT, The Nutrition Literacy Assessment Instrument for Italian people; NLit-Br, The Nutrition Literacy Assessment Instrument for Brazilians; CHI-NLit, The Chinese Version of the Nutrition Literacy Assessment Instrument; ANLS, The Adolescent Nutritional Literacy Scale; EINLA, The Evaluation Instrument of Nutrition Literacy on Adults; e-NutLiT, The Electronic Nutrition Literacy Tool; CNL-E, The Adolescent Critical Nutrition Literacy Scale; FNLQ-SC, The Nutrition Literacy Scale for Chinese School-Aged Children; NLQ-E, The Nutrition Literacy Questionnaire for the Elderly; NLAI-P, The Nutrition Literacy Assessment Instrument for Pregnant Women; FNLQ, The Nutrition Literacy Scale for the General Chinese Population; NLAI-L, The Nutrition Literacy Assessment Instrument for Chinese Lactating Women; NLQ-PSC, The Nutrition Literacy Questionnaire for Preschool Children.

**Table 3:** Summary of studies examining the relationship between nutritional literacy, health behaviors, and health outcomes.

Research Areas	Tool Name	Country/Region of Origin	Target Audience	Statistical Methods	Key Findings	Literature Cited
Dietary behavior	CNL; NLS; EINLA; NLit-S; NLAI-P	Palestine, China, Turkey; Azerbaijan; Iran, Chile	Adults; Preschoolers; Adolescents; Children; College students; Pregnant women	Pearson correlation analysis; factor analysis; mediation effect testing; independent samples *t*-test; one-way analysis of variance	Nutrition literacy is positively correlated with dietary behavior; Parenting styles mediate the conversion of nutrition literacy into dietary behavior among preschool children.	(Al Tell et al., 2023 [39]; Ningning & Wenguang, 2023 [40]; Mammadova et al., 2025 [41]; Alpat Yavaş et al., 2024 [42]; Yilmazel & Bozdogan, 2021 [43]; Li et al., 2024 [44])
Understanding and Using Nutrition Labels	CNL; NLS; NVS	China; Turkey; Palestine; Hong Kong; China	Adults; Adolescents	Chi-square test; Regression Analysis; One-way analysis of variance	The higher the level of nutrition literacy, the stronger the ability to understand and utilize nutrition labels.	(Yang et al., 2024 [45]; Yilmazel & Bozdogan, 2021 [43]; Al Tell et al., 2023 [39]; Law et al., 2019 [46])
Nutritional and Health Status	NLS; NLit-S; NLQ-E	China; Chile	Adolescents; College students; Older adults	Chi-square test; Regression Analysis; independent samples *t*-test; ANOVA; Structural equation modeling	Nutrition literacy is positively associated with nutritional and health status and is influenced by nutrition attitude, social support, daily living ability, and self-efficacy.	(Li et al., 2022 [47]; Herrera et al., 2021 [48]; Mammadova et al., 2025 [41]; Maheri et al., 2022 [49]; Alpat Yavaş et al., 2024 [42]; Liu et al., 2025 [50] Liu et al., 2024 [51])
Nutrition Information Access	CNL	Palestine	Adults	Pearson correlation analysis	Nutrition literacy is positively correlated with the ability to access nutrition information.	(Al Tell et al., 2023 [39])
Sleep	NLS	China	Adolescents	Chi-square test; Regression Analysis	Nutrition literacy is positively correlated with sleep quality, and nutrition literacy interventions yield spillover effects on health behaviors.	(Xu et al., 2024 [52])
Dietary Patterns Among Adults with Chronic Diseases	NLit	United States	Chronic disease patients	Pearson correlation analysis; Regression Analysis	Nutrition literacy is positively correlated with healthy dietary patterns among individuals with chronic diseases.	(Taylor et al., 2019 [4])
Quality of Life	EINLA	Turkey	Adolescents; College students	independent samples *t*-test; ANOVA; Pearson correlation analysis; Regression Analysis	Nutritional literacy is positively correlated with quality of life, simultaneously enhancing subjective health levels and life satisfaction.	(Baş et al., 2024 [53]; Aslan Ceylan et al., 2024 [54])

Note: Compiled by the authors based on relevant literature. Abbreviations are defined at the end of the manuscript.

## 4. Discussion

### 4.1. Evolution and Development of Nutrition Literacy Scales

This review systematically traces the evolution of nutrition literacy scales from 1963 to 2025, as well as their current applications, highlighting the developmental trajectory of this field. This review covers initial conceptual exploration to methodological refinement and unidimensional assessment to multidimensional adaptation. The developmental journey of nutrition literacy scales fundamentally reflects the systematic evolution of the concept of “nutrition literacy” from functional skills towards a multidimensional, comprehensive competency. From early tools anchored within health literacy frameworks focused on measuring functional literacy skills, the concept has progressively expanded to encompass knowledge, skills, and behavioral application across multiple dimensions. Both its theoretical foundations and measurement paradigms have undergone significant evolution. Early instruments, exemplified by the NVS and NLS, predominantly employed single-dimensional structures, with measurement concentrating on functional capacities for healthy eating, such as reading comprehension and numerical calculation. Notably, the NLS was the first to explicitly define “nutrition literacy” as a relatively independent measurement target at the scale level, marking the concept’s differentiation from the general health literacy framework. Following the introduction of Nutbeam’s three-tier health literacy model [12], a three-dimensional framework encompassing functional, interactive, and critical dimensions was established in the NLQ (Nutrition Literacy Questionnaire). This shifted the focus from assessing singular healthy eating skills towards integrating multi-level competencies. Subsequently, the dimensions were expanded to six in the NLAI, and multiple localized versions of this tool were generated, progressively establishing a multidimensional assessment paradigm integrating knowledge, skills, and application. This laid the methodological foundation for subsequent tool development. Since 2014, the development of nutrition literacy scales has followed multiple paths. First, the dimension integration pathway, exemplified by the EINLA, incorporates knowledge comprehension and practical skills into a systematic, coherent measurement framework, emphasizing structural integrity and theoretical consistency. Second, the specialized development approach, exemplified by the e-NutLit (Electronic Nutrition Literacy Tool) and CNL-E (Adolescent Critical Nutrition Literacy Scale), focuses on in-depth development for specific application scenarios or population needs. This approach sacrifices dimensional completeness to some extent in exchange for measurement precision within domains. Third, Chinese scholars have systematically constructed multiple versions of localized tools tailored to the Chinese context based on the National Dietary Guidelines framework. Target populations include preschool children, school-age children, the elderly, pregnant and lactating women, and the general adult population. While maintaining stability in the core dimensional framework, each version features differentiated designs according to the cognitive development levels and life contexts of the target groups, reflecting a dynamic equilibrium between structural consistency and contextual adaptability.

### 4.2. Measurement Methods and Scoring Logic

Regarding scoring methodologies, existing scales’ scoring logic can be categorized into two complementary systems: objective testing and subjective self-assessment. Objective testing typically features accuracy rates or task completion quality as metrics, emphasizing standardization and cross-group comparability, yet it remains highly dependent on examinees’ linguistic proficiency and cultural background. Subjective self-assessment predominantly features Likert scales, enabling latent traits such as attitudinal inclinations and critical thinking to be captured, though it remains susceptible to interference from social desirability effects. In recent years, method integration has emerged as a significant trend. For example, the NLQ-JP (Nutrition Literacy Questionnaire Japanese) combines knowledge-based questions, task-oriented comprehension items, and Likert scales, achieving structural fusion of objective testing and subjective evaluation, extending the measurement scope from basic comprehension of nutritional information to a critical assessment of diverse nutritional information within complex scenarios. This demonstrates a simultaneous expansion of both the measurement boundaries and theoretical implications.

### 4.3. Population Applicability, Cultural Adaptation, and Tool Reliability

Regarding population applicability and geographical coverage, nutrition literacy scales have progressively expanded beyond general adult populations to include specific groups such as chronic disease patients, the elderly, adolescents, pregnant and lactating women, and preschool children. Application has expanded from developed areas like Europe, America, and Japan to encompass developing nations, including China, Turkey, Brazil, and Uganda. During localization, research priorities have transcended mere linguistic translation, shifting towards systematic adaptations such as food example selection, nutrition labeling formats, and dietary cultural norms. This ensures the content and ecological validity of the tools across diverse cultural contexts. Overall, nutritional literacy measurement systems continue to evolve towards multidimensional structures, integrated methodologies, and cultural adaptation.

Although the target populations and countries in which nutrition literacy scales are applied have gradually expanded, the reliability of existing measurement tools still varies across studies. Some scales, such as the NLAI, NLS, NLQ, CNL, and several adapted versions, have shown strong internal consistency, with Cronbach’s α values generally exceeding 0.80. In contrast, some tools have reported relatively low internal consistency, including the ANLS (Adolescent Nutritional Literacy Scale) (α = 0.59), FNLQ-SC (Nutrition Literacy Scale for Chinese School-Aged Children) (α = 0.698), NLQ-E (Nutrition Literacy Questionnaire for the Elderly) (α = 0.678), and NLQ-PSC (Nutrition Literacy Questionnaire for Preschool Children) (α = 0.660 and 0.628 depending on the respondent group). These findings suggest that the reliability of certain scales may be limited when applied to specific populations or cultural contexts, and the results obtained from these tools should therefore be interpreted with caution. Overall, nutrition literacy measurement systems continue to evolve to achieve more reliable measurement performance. Future research should further distinguish between relatively stable scales and those requiring additional validation, particularly when these tools are applied across different age groups, health conditions, and cultural environments.

### 4.4. Application Scenarios and Future Research Directions

As the scale system has progressively matured, its applications have continually expanded. Since 2015, the volume of applied research utilizing the nutrition literacy scales has grown significantly, with research themes and methodologies undergoing phased evolution. Early studies focused on chronic disease populations, employing multidimensional scales such as the NLit to examine the association between nutrition literacy and overall dietary patterns. These investigations preliminarily uncovered a positive relationship between the two, providing an empirical foundation for subsequent exploration of the behavioral conversion mechanisms of nutrition literacy. At that time, the global burden of chronic diseases continued to rise, and nutritional literacy was viewed as a potential pathway to enhance patients’ self-management capabilities. Research designs primarily employed descriptive correlation analyses. As food labeling regulations gradually matured in countries such as China and Turkey, the policy significance of nutrition labeling became increasingly prominent. Consequently, the focus of related research shifted to individuals’ comprehension and application of label information. The NVS, NLS, and CNL emerged as core tools during this phase, with statistical methods evolving from simple correlation analyses to regression modeling and group comparisons. Research consistently demonstrated that higher levels of nutritional literacy were correlated with improved label interpretation and healthier purchasing decisions.

Entering the 2020s, against the backdrop of a global paradigm shift towards behavioral interventions and lifecycle management for promoting health, research perspectives broadened further. Lifestyle factors such as parenting practices, sleep quality, and physical activity were incorporated into analytical frameworks, revealing the spillover effects of nutritional literacy. In addition, specific populations, including children, adolescents, and pregnant women, garnered increased attention. The introduction of mediation models and factor analysis propelled research beyond descriptive assessments towards deeper explorations of nutritional literacy’s mechanisms of action. Regarding tools, scales such as EINLA and NLit-S, which encompass knowledge, attitude, and practice dimensions, have increasingly been prioritized to more effectively capture the conversion from cognition to behavior.

Overall, the existing evidence demonstrates relatively consistent conclusions across multiple countries and populations: enhanced nutrition literacy facilitates the translation of nutritional information into concrete dietary choices and health practices. Nevertheless, existing research predominantly relies on cross-sectional designs, with causal directionality yet to be fully validated. Samples also remain concentrated in high-income nations and specific age cohorts. Future studies should incorporate longitudinal, multicenter designs and intervention trials, alongside digital assessment tools, to deepen our understanding of the causal mechanisms through which nutritional literacy influences health behaviors.

## 5. Conclusions

This scoping review provides a comprehensive overview of the evolution, measurement characteristics, applications, and limitations of nutrition literacy scales. Overall, methods for measuring nutrition literacy have evolved from early functional tools derived from health literacy frameworks to more independent and multidimensional instruments covering knowledge, skills, application, and critical literacy. Target populations and geographical contexts have also expanded from general adults and developed countries to specific populations and developing regions.

The current evidence suggests that nutrition literacy scales have been increasingly applied in nutrition education, chronic disease prevention, dietary behavior assessment, and health promotion. The rapid development of localized tools in China has further enriched the evidence beyond Western contexts and provides useful experience for other developing countries. However, challenges remain, including inconsistent dimensional structures, insufficient standardization, variable reliability across tools, limited cultural adaptation, and the predominance of cross-sectional study designs.

Future studies should establish more unified evaluation frameworks, strengthen reliability and validity testing, enhance cultural and population-specific adaptation, and promote longitudinal and intervention studies. Continued improvement of nutrition literacy measurement tools will support better assessment, comparison, and intervention, thereby contributing to healthier dietary behaviors and disease prevention.

## Data Availability

No new data were created or analyzed in this study. Data Sharing is not applicable to this article.

## Abbreviations

The following abbreviations are used in this manuscript:

NVS	The Newest Vital Sign
NLS	The Nutritional Literacy Scale
Spanish-NLS	The Spanish Nutrition Literacy Scale
NLS-Gr	The Greek Version of the Nutrition Literacy Scale
NLQ	The Nutrition Literacy Questionnaire
NLQ-JP	The Nutrition Literacy Questionnaire for Japanese
CNL	The Critical Nutrition Literacy Scale
NLAI	The Nutrition Literacy Assessment Instrument
NLit-BCa	The Nutrition Literacy Assessment Instrument for Breast Cancer
NLit-P	The Nutrition Literacy Assessment Instrument for Parents
NLit	The Nutrition Literacy Assessment Instrument
NLit-S	The Nutrition Literacy Assessment Instrument in Spanish
NLit-IT	The Nutrition Literacy Assessment Instrument for Italian people
NLit-Br	The Nutrition Literacy Assessment Instrument for Brazilians
CHI-NLit	The Chinese Version of the Nutrition Literacy Assessment Instrument
ANLS	The Adolescent Nutritional Literacy Scale
EINLA	The Evaluation Instrument of Nutrition Literacy on Adults
e-NutLit	The Electronic Nutrition Literacy Tool
CNL-E	The Adolescent Critical Nutrition Literacy Scale
FNLQ-SC	The Nutrition Literacy Scale for Chinese School-Aged Children
NLQ-E	The Nutrition Literacy Questionnaire for the Elderly
NLAI-P	The Nutrition Literacy Assessment Instrument for Pregnant Women
FNLQ	The Nutrition Literacy Scale for the General Chinese Population
NLAI-L	The Nutrition Literacy Assessment Instrument for Chinese Lactating Women
NLQ-PSC	The Nutrition Literacy Questionnaire for Preschool Children

## References

[1] Gakidou E., Afshin A., Abajobir A.A., Abate K.H., Abbafati C., Abbas K.M., Abd-Allah F., Abdulle A.M., Abera S.F., Aboyans V. (2017). Global, Regional, and National Comparative Risk Assessment of 84 Behavioural, Environmental and Occupational, and Metabolic Risks or Clusters of Risks, 1990–2016: A Systematic Analysis for the Global Burden of Disease Study 2016. Lancet.

[2] World Health Organization (2025). Tracking Progress on the Implementation of the Global Oral Health Action Plan 2023–2030: Baseline Report.

[3] Willett W., Rockström J., Loken B., Springmann M., Lang T., Vermeulen S., Garnett T., Tilman D., DeClerck F., Wood A. (2019). Food in the Anthropocene: The EAT–Lancet Commission on Healthy Diets from Sustainable Food Systems. Lancet.

[4] Taylor M.K., Sullivan D.K., Ellerbeck E.F., Gajewski B.J., Gibbs H.D. (2019). Nutrition Literacy Predicts Adherence to Healthy/Unhealthy Diet Patterns in Adults with a Nutrition-Related Chronic Condition. Public Health Nutr..

[5] Haddaway N.R., Page M.J., Pritchard C.C., McGuinness L.A. (2022). PRISMA2020: An R Package and Shiny App for Producing PRISMA 2020-Compliant Flow Diagrams, with Interactivity for Optimised Digital Transparency and Open Synthesis. Campbell Syst. Rev..

[6] Blitstein J.L., Evans W.D. (2006). Use of Nutrition Facts Panels among Adults Who Make Household Food Purchasing Decisions. J. Nutr. Educ. Behav..

[7] Neuhauser L., Rothschild R., Rodríguez F.M. (2007). MyPyramid.Gov: Assessment of Literacy, Cultural and Linguistic Factors in the USDA Food Pyramid Web Site. J. Nutr. Educ. Behav..

[8] Silk K.J., Sherry J., Winn B., Keesecker N., Horodynski M.A., Sayir A. (2008). Increasing Nutrition Literacy: Testing the Effectiveness of Print, Web Site, and Game Modalities. J. Nutr. Educ. Behav..

[9] Watson W.L., Chapman K., King L., Kelly B., Hughes C., Louie J.C.Y., Crawford J., Gill T.P. (2013). How Well Do Australian Shoppers Understand Energy Terms on Food Labels?. Public Health Nutr..

[10] Zoellner J., Connell C., Bounds W., Crook L., Yadrick K. (2009). Nutrition Literacy Status and Preferred Nutrition Communication Channels among Adults in the Lower Mississippi Delta. Prev. Chronic Dis..

[11] Guttersrud Ø., Dalane J.Ø., Pettersen S. (2014). Improving Measurement in Nutrition Literacy Research Using Rasch Modelling: Examining Construct Validity of Stage-Specific ‘Critical Nutrition Literacy’ Scales. Public Health Nutr..

[12] Nutbeam D. (2000). Health Literacy as a Public Health Goal: A Challenge for Contemporary Health Education and Communication Strategies into the 21st Century. Health Promot. Int..

[13] Weiss B.D., Mays M.Z., Martz W., Castro K.M., DeWalt D.A., Pignone M.P., Mockbee J., Hale F.A. (2005). Quick Assessment of Literacy in Primary Care: The Newest Vital Sign. Ann. Fam. Med..

[14] Diamond J.J. (2007). Development of a Reliable and Construct Valid Measure of Nutritional Literacy in Adults. Nutr. J..

[15] Coffman M.J., La-Rocque S. (2012). Development and Testing of the Spanish Nutrition Literacy Scale. Hisp. Health Care Int..

[16] Michou M., Panagiotakos D.B., Costarelli V. (2019). Development & Validation of the Greek Version of the Nutrition Literacy Scale. Mediterr. J. Nutr. Metab..

[17] Kjøllesdal J.G. (2009). Nutrition Literacy: Utvikling og Utprøving av et Spørreskjema Som Måler Grader av Nutrition Literacy. Master’s Thesis.

[18] Aihara Y., Minai J. (2011). Barriers and Catalysts of Nutrition Literacy among Elderly Japanese People. Health Promot. Int..

[19] Blegen H.H. (2011). Nutrition Literacy hos 10. Klasseelever i en Østlandskommune. Master’s Thesis.

[20] Gibbs H.D. (2012). Nutrition Literacy: Foundations and Development of an Instrument for Assessment. Ph.D. Dissertation.

[21] Gibbs H.D., Ellerbeck E.F., Befort C., Gajewski B., Kennett A.R., Yu Q., Christifano D., Sullivan D.K. (2016). Measuring Nutrition Literacy in Breast Cancer Patients: Development of a Novel Instrument. J. Cancer Educ..

[22] Gibbs H.D., Kennett A.R., Kerling E.H., Yu Q., Gajewski B., Ptomey L.T., Sullivan D.K. (2016). Assessing the Nutrition Literacy of Parents and Its Relationship with Child Diet Quality. J. Nutr. Educ. Behav..

[23] Gibbs H.D., Harvey S., Owens S., Boyle D., Sullivan D.K. (2017). Engaging Experts and Patients to Refine the Nutrition Literacy Assessment Instrument. BMC Nutr..

[24] Gibbs H.D., Camargo J.M.T.B., Owens S., Gajewski B., Cupertino A.P. (2018). Measuring Nutrition Literacy in Spanish-Speaking Latinos: An Exploratory Validation Study. J. Immigr. Minor. Health.

[25] Vettori V., Lorini C., Gibbs H.D., Sofi F., Lastrucci V., Sartor G., Fulvi I., Giorgetti D., Cavallo G., Bonaccorsi G. (2021). The Nutrition Literacy Assessment Instrument for Italian Subjects, NLit-IT: Exploring Validity and Reliability. Int. J. Environ. Res. Public Health.

[26] Sarkis L.B.d.S., Teruel-Camargo J., Gibbs H.D., Nakano E.Y., Ginani V.C., de Aguiar A.S., Chaves C.d.S., Zandonadi R.P., Bastos M.G. (2022). The Nutrition Literacy Assessment Instrument for Brazilians, NLit-Br: An Exploratory Cross-Cultural Validity Study. Nutrients.

[27] Zhang J., Li D., Yan J., Yang J., Sun J., Liu Y., Xia Y., Cao H., Hua J., Zhang F. (2025). Factors Influencing Nutrition Literacy in Patients of Colorectal Cancer: A Cross-Sectional Study. Front. Nutr..

[28] Bari N.N. (2012). Nutrition Literacy Status of Adolescent Students in Kampala District, Uganda. Master’s Thesis.

[29] Cesur B. (2014). Sivas İl Merkezi Yetişkin Nüfusta Beslenme Okuryazarlığı Durumu ve Yaşam Kalitesi ile İlişkisi. Ph.D. Dissertation.

[30] Ringland E.M., Gifford J.A., Denyer G.S., Thai D., Franklin J.L., Stevenson M.M., Prvan T., O’connor H.T. (2016). Evaluation of an Electronic Tool to Assess Food Label Literacy in Adult Australians: A Pilot Study. Nutr. Diet..

[31] Naigaga D.A., Pettersen K.S., Henjum S., Guttersrud Ø. (2018). Assessing Adolescents’ Perceived Proficiency in Critically Evaluating Nutrition Information. Int. J. Behav. Nutr. Phys. Act..

[32] Liu T., Su X., Li N., Sun J., Ma G., Zhu W. (2021). Development and Validation of a Food and Nutrition Literacy Questionnaire for Chinese School-Age Children. PLoS ONE.

[33] Aihemaitijiang S., Ye C., Halimulati M., Huang X., Wang R., Zhang Z. (2022). Development and Validation of Nutrition Literacy Questionnaire for the Chinese Elderly. Nutrients.

[34] Zhou Y., Lyu Y., Zhao R., Shi H., Ye W., Wen Z., Li R., Xu Y. (2022). Development and Validation of Nutrition Literacy Assessment Instrument for Chinese Pregnant Women. Nutrients.

[35] Zhang Y., Zhang Z., Xu M., Aihemaitijiang S., Ye C., Zhu W., Ma G. (2022). Development and Validation of a Food and Nutrition Literacy Questionnaire for Chinese Adults. Nutrients.

[36] Zhang Y., Sun Q., Zhang M., Mo G., Liu H. (2022). Nutrition Literacy Measurement Tool with Multiple Features for Chinese Adults. Food Nutr. Bull..

[37] Li Z., Zhou Y., Tan Y., Zhu X., Liu W., Chen Y., Qin Y., Li R., Yu L., Zhao R. (2023). Development and Validation of Nutrition Literacy Assessment Instrument for Chinese Lactating Women: A Preliminary Study. Nutrients.

[38] Wen J., Zhang X., Yin X., Ma G., Wang J. (2025). Development and Validation of Nutrition Literacy Questionnaire for Chinese Pre-School Children. Nutrients.

[39] Al Tell M., Natour N., Alshawish E., Badrasawi M. (2023). The Relationship between Nutrition Literacy and Nutrition Information Seeking Attitudes and Healthy Eating Patterns among a Group of Palestinians. BMC Public Health.

[40] Ningning W., Wenguang C. (2023). Influence of Family Parenting Style on the Formation of Eating Behaviors and Habits in Preschool Children: The Mediating Role of Quality of Life and Nutritional Knowledge. PLoS ONE.

[41] Mammadova S., Tezol O., Temel G. (2025). Investigating the Correlations between Nutrition Literacy of Mothers and Offspring Physical Growth and Development, Dietary Diversity and Quality, and Vitamin Levels. North. Clin. İstanbul.

[42] Alpat Yavaş İ., Guney-Coskun M., Saleki N., Sezer Karataş F.E., Keskin E. (2024). Nutrition Literacy and Its Relationship with Diet Quality and Quality of Life among White-Collar Employees: A Study from Türkiye. BMC Public Health.

[43] Yilmazel G., Bozdogan S. (2021). Nutrition Literacy, Dietary Habits and Food Label Use among Turkish Adolescents. Prog. Nutr..

[44] Li Q., Piaseu N., Phumonsakul S., Thadakant S. (2024). Effects of a Comprehensive Dietary Intervention Program, Promoting Nutrition Literacy, Eating Behavior, Dietary Quality, and Gestational Weight Gain in Chinese Urban Women with Normal Body Mass Index during Pregnancy. Nutrients.

[45] Yang L., Cui Y., Du J., Liu Z., Duan Y., Qi Q., Liu H., Zhang M. (2024). Association between Nutritional Literacy and Nutrition Label Use in Chinese Community Residents. Front. Nutr..

[46] Law Q.P.S., Yau A.H.Y., Chung J.W.Y. (2019). Chinese Adults’ Nutrition Label Literacy in Hong Kong: Implications for Nurses. Nurs. Health Sci..

[47] Li S., Zhu Y., Zeng M., Li Z., Zeng H., Shi Z., Zhao Y. (2022). Association Between Nutrition Literacy and Overweight/Obesity of Adolescents: A Cross–Sectional Study in Chongqing, China. Front. Nutr..

[48] Herrera P., Gálvez P., Cuevas C., Sanhueza D. (2021). Una aproximación a la alfabetización nutricional, evaluación del estado nutricional y calidad de la alimentación en una muestra a conveniencia de mujeres de comunas con pobreza multidimensional. Rev. Chil. Nutr..

[49] Maheri M., Bidar M., Farrokh-Eslamlou H., Sadaghianifar A. (2022). Evaluation of Anthropometric Indices and Their Relationship with Maternal Nutritional Literacy and Selected Socio-Economic and Demographic Variables among Children under 5 Years Old. Ital. J. Pediatr..

[50] Liu S., Fan X., Jiang L., Liu T. (2025). Factors Influencing Nutritional Literacy among Rural Older Adults: A Cross-Sectional Survey Based on the Theory of Planned Behavior. Front. Nutr..

[51] Liu Y., Zhang L., Xu K., Ding Y., Li F., Zhang T. (2024). Cross-Cultural Adaptation and Validation of the Short Nutritional Literacy Scale for Young Adults (18–35 years) and Analysis of the Influencing Factors. BMC Public Health.

[52] Xu Q., Hu Z., Zeng M., Su Y., Jiang K., Li S., Li Z., Fu L., Shi Z., Sharma M. (2024). Relationships among Sleep Time, Physical Activity Time, Screen Time, and Nutrition Literacy of Adolescents: A Cross-Sectional Study in Chongqing, China. Nutrients.

[53] Baş D., Tontaş E., Kavuşan K.A., Seçkiner S., Sakar Schoinas E., Kayak S. (2024). Relationship between Nutritional Literacy and Quality of Life in Turkish Adults: A Cross-Sectional Survey. J. Health Lit..

[54] Aslan Ceylan J., Bayindir Gumus A. (2024). Nutrition Literacy and Quality of Life of University Students: Evidence from A Cross-Sectional Survey. J. Health Lit..

